# Hardness, plasticity, and oil binding capacity of binary mixtures of natural waxes in oliveoil

**DOI:** 10.1016/j.crfs.2022.06.002

**Published:** 2022-06-13

**Authors:** Saeed M. Ghazani, Stacie Dobson, Alejandro G. Marangoni

**Affiliations:** Department of Food Science, University of Guelph, Guelph, ON, N1G 2W1, Canada

**Keywords:** Natural wax, Oleogel, Hardness, Plasticity, Oil-binding capacity, Mixing behavior

## Abstract

In this study, olive oil oleogels structured with less than 4% binary blends of sunflower wax (SFW), rice bran wax (RBW), candelilla wax (CDW), and beeswax (BW) were characterized. Among the different binary wax oleogels, samples structured with 3% (w/w) of binary mixtures of SFW and RBW, as well as binary mixtures of CDW and BW, displayed a high oil binding capacity relative to any other mixtures. Moreover, in some binary wax oleogels, back extrusion hardness and elastic constant were significantly higher than that of oleogels prepared using the individual waxes. This was interpreted as a synergism between these waxes. Image analysis of oleogel brightfield micrographs indicated that the samples with a higher elastic constant had a lower box-counting fractal dimension and larger crystals, suggesting that this increase in the elastic constant was a consequence of the lower fractal dimension of the wax crystal network, in agreement with established fractal structural-mechanical models for van der Waals colloidal networks. The crystal structure of the individual waxes and their blends showed orthorhombic perpendicular subcell packing arrangements, which did not change upon mixing, suggesting this length scale did not play a role in the observed synergism. The melting point of binary mixtures of waxes in olive oil was in the range of 43.2 °C to 67.4 °C and pseudo-ideal mixing behavior was observed. The hardness and plasticity (brittleness) of the 2% and 3% binary wax mixtures in olive oil characterized using back extrusion, were similar to those of a commercial soft margarine, suggesting a potential use of the wax oleogels as margarine or spread replacers.

## Highlights

• Oleogels with binary mixtures of sunflower wax and rice bran wax had a high oil binding capacity.
• Binary mixture wax oleogels were harder than those structured using individual waxes.
• The crystal structure and polymorphism of individual waxes and their blends in olive oil were similar.
• A blend of SFW and RBW in olive oil at 3% levels structured olive oil resembling margarine texture.

## Graphical abstract

**Figure:**
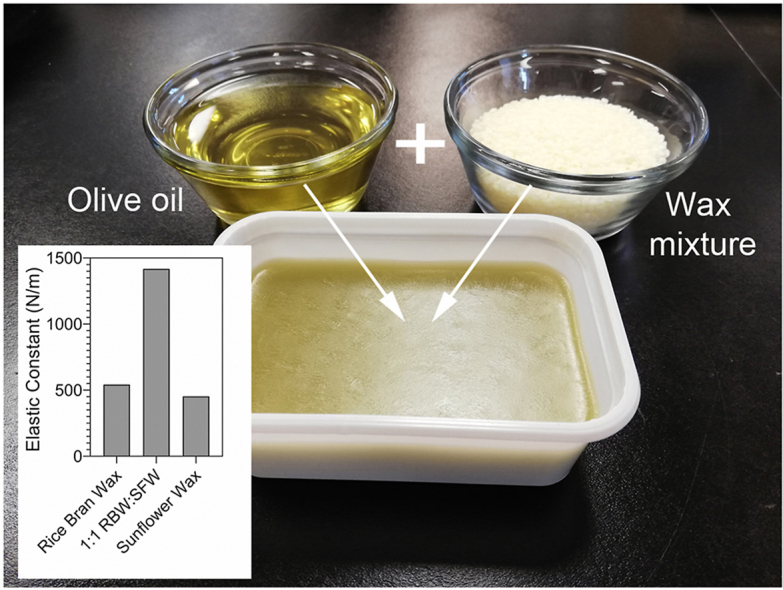

## 1 Introduction

Traditionally, a certain amount of solid fat is required in margarine and shortening manufacture to stabilize emulsions, achieve a desirable hardness, plasticity, and create a desirable melting profile. On an industrial scale, non-hydrogenated, natural sources of solid fats are limited to palm oil and its derivatives, cocoa butter, plus some exotic butters such as sal fat, shea stearin, illipe butter, mango fat, and others. Complicating matters further, palm oil is not considered an ideal option today, due to its lack of sustainability related to tropical rainforest destruction and loss of biodiversity (Austin et al., 2017). On the other hand, exotic butters are not widely available on a large scale for industrial purposes (Salas et al., 2009). This has forced the food industry to use hydrogenation (partial or full hydrogenation), alongside interesterification (chemical or enzymatic), to modify the molecular composition of vegetable oils to obtain fats with desirable physical and functional properties.

Previous studies have shown that hydrogenation and chemical interesterification not only increase processing costs but also lead to the removal of healthy minor components from oils such as natural antioxidants, phytosterols, and polyphenols (Azadmard-Damirchi and Dutta, 2008). Moreover, *trans*-fatty acids, 3-monochloropropane-1,2-diol (3-MCPD), and glycidyl esters are usually produced as a result of the hydrogenation and chemical interesterification (Mensink and Katan, 1990; Gibon et al. 2018). So, finding new oil-structuring alternatives to conventional fat modification processes is appealing to both food manufacturers and customers.

Recently, the use of low concentrations of food-grade oleogelators (<5% w/w) was introduced to the food industry in order to structure and entrap liquid oils into a fat-like structure to create spreads and shortenings (Blake et al., 2018; Patel, 2015; Soleimanian et al., 2020). Of the many gelators available, natural waxes, including sunflower wax (SFW), rice bran wax (RBW), candelilla wax (CDW), carnauba wax (CRW), and beeswax (BW) are some of the most promising. All of the above-mentioned waxes have gained GRAS (Generally Regarded as Safe) status approved by the FDA for use in food products, except sunflower wax, which has not been approved yet. After melting and cooling a small amount of natural waxes into the liquid oil, they crystallize to form a 3-dimensional crystal network which provides solid structure and oil binding characteristics. Natural wax oleogelators are complex mixtures of different compounds, including esters of long-chain aliphatic fatty acids and fatty alcohols, free fatty acids, fatty alcohols, hydrocarbons, and other minor components. For example, BW is mainly a complex blend of long-chain aliphatic monohydric alcohols with chain lengths from 24 to 36 esterified to long aliphatic chain fatty acids with up to 36 carbon atoms, as well as 18 carbon hydroxylated and non-hydroxylated free fatty acids (Martins et al., 2016). However, the chemical composition of some natural waxes is more complicated, for instance, CDW contains about 50% n-alkanes with chain lengths of 29–33 carbons, 20–29% long aliphatic chain esters, 12–14% alcohols and sterols, and 7–9% free fatty acids (Toro-Vazquez, Morales-Rueda, Dibildox-Alvarado, Charó-Alonso, Alonzo-Macias & González-Chávez, 2007).

The digestibility of wax oleogels and their ability to encapsulate and deliver of nutraceuticals have also been studied by many researchers. For example, in 2020, Calligaris et al. showed that the digestibility of sunflower oil enriched with curcumin gelled using 5% RBW was greatly affected by oleogel structure. They showed the extent of lipolysis was higher for RBW oleogels compared to a 5% (w/w) saturated monoglycerides oleogels. Moreover, a recently published report showed that the amount of natural bio-active components (tocopherols and polyphenols) in extra virgin olive oil gelled with 10% (w/w) RBW was higher compared to 10% (w/w) monoglycerides, γ-oryzanol and β-sitosterol, or ethylcellulose (EC) oleogels. (Alongi et al., 2022).

In the past few years, many attempts have been made to formulate margarine and shortening using wax organogels (Yılmaz and Öğütcü, 2014 and 2015, Limpimwong et al., 2017 and Hwang and Winkler-Moser, 2020). However, in a US patent published in 2003, the inventors disclosed a composition that comprises a blend of vegetable oil and an individual natural wax component from a plant species including SFW, RBW, and corn oil wax (COW) to formulate low saturate, low *trans* fatty acid food products such as spreads, margarines, and shortenings. They reported the amount of plant-derived wax component was at least about 0.1% (w/w) to about 30% (w/w) of the total composition. Based on the amount of plant-based wax in the blends, the range of melting points was from 5 °C to 75 °C (Loh et al., 2003).

Wax-based spreads and margarines would contain very low amounts of saturated fatty acids (depends on the amount of wax and saturated fatty acids in plant oil used in the formula) and zero levels of *trans* isomers. Previous studies also showed the health benefits of consuming wax oleogel margarines compared to commercial ones. For example, in 2017, Limpimwong et al. produced a margarine containing 5% (w/w) RBW in rice bran oil (RBW-RBO). Then, they compared the lipid digestibility of the oleogel margarine to that of a commercial margarine by feeding rats for four weeks. They showed that the consumption of oleogel margarine caused a decrease in adipose tissue accumulation, triacylglycerol content in blood serum, and total cholesterol levels in the liver. They concluded RBW-RBO oleogel margarine potentially could be utilized to make a spread with healthier properties and lower caloric content. Wax oleogels can also prevent fatty acid oxidation. In a research study that was published in 2015, Öğütcü, Arifoğlu & Yılmaz showed no significant changes in texture or oxidation status of a spreadable cod liver oil (CLO) wax oleogels using either BW or CRW at different concentrations after a 90-day storage at 4 °C and 20 °C. They concluded that gelation of CLO using BW or CRW could be a way to mask the flavor of fish oil, increase oxidative stability, and provide new functionalities such as spreadability and hardness in the final product. In 2014, Yılmaz and Öğütcü studied and compared oxidative stability (peroxide value and free fatty acid content analysis), physical properties (melting profile, solid fat content, and crystal size), textural properties (hardness and stickiness), and sensory properties of margarine and butter substitutes made using 5% (w/w) BW and SFW oleogels in hazelnut oil, olive oil, and sunflower oil. They showed the textural properties of 3% SW and 7% BW wax oleogels resembled those of a breakfast margarine. In 2013, Hwang et al. used 2–6% (w/w) SFW, RBW, and CDW in soybean oil to prepare an oleogel margarine containing 80% wax oleogel, 0.2% mono- and di-acylglycerol, and a water 19.8% phase composed of 19.46% skim milk, 0.1% salt, 0.1% lecithin, 0.03% citric acid, 0.0075% calcium disodium EDTA and 0.1% potassium sorbate. The authors reported that a phase separation was observed in the emulsion formulated with CDW in the oil phase. The hardness of the oleogel margarine made with RBW was lower than that of commercial margarine, while the oleogel margarine containing 2–6% SFW showed similar hardness to a commercial margarine containing 18–30% hydrogenated soybean oil. Similar studies have also shown the great potential of using wax oleogels to produce margarine, shortening, and spreads compared to commercial margarines and spreads with value-added properties such as higher thermal and oxidative stability and improved physical, textural, and sensory characteristics (da Silva et al., 2018; Yılmaz and Öğütcü, 2014).

While most of the work on wax oleogels focused mainly on the use of single waxes in edible oil, Jana and Martini (2016a,b) studied the phase behavior of different binary mixtures of SFW, BW, RBW, and paraffin wax (PW) using differential scanning calorimetry (DSC) and polarized light microscopy (PLM). They reported eutectic phase behavior for binary mixtures of BW-PW, SFW-PW, SFW-BW, and RBW-BW, while the RBW-SFW mixture exhibited ideal solid solution behavior. Jana and Martini (2016a) demonstrated that when two waxes are made up of different components, they tend to form eutectic or monotectic mixtures, and these mixed compositions tend to form smaller and denser crystal morphologies. Following this study, Jana and Martini (2016b), studied the physical properties of binary mixtures of 2.5% (w/w) SFW, RBW, and BW in soybean oil. They concluded that blending waxes did not result in linear changes in physical properties and showed antagonism and synergism effects in the different wax blends in soybean oil. In a recently published paper in 2019, Winkler-Moser et al. showed eutectic melting properties for the CDW-BW mixtures at the ratio of 30:70 (w/w). However, Shi et al. (2021) studied the oleogelation and structural properties of 5% binary wax mixtures of containing BW, CRW, RBW, and China lacquer wax (ZLW). They showed that the binary oleogels exhibited monotectic mixing behavior.

Previous research has shown that the interaction between waxes and other components in the oil phase, such as mono-, di- and triacylglycerols, lecithin, and synthetic emulsifiers, can also lead to changes in crystallization temperature, crystal structure, and physical properties of oleogel mixtures. For example, Chopin-Doroteo, Morales-Rueda, Dibildox-Alvarado, Charó-Alonso, de la Peña-Gil, and Toro-Vazquez (2011) showed that the addition of 1% tripalmitin to 3% CDW in vegetable oils resulted in increased elasticity of wax oleogel mixtures. In another research in 2017, Limpimwong et al. showed that the crystallization and gelation properties of binary mixtures of 5% w/w of the high-melting SFW or RBW wax with the low-melting berry wax in rice bran oil resulted in an improvement in the rheological properties and an increase in the hardness of the wax oleogels. These authors reported a sintering effect (solid bridges) between the crystals of berry wax and SFW or RBW wax, which led to an increase in the cohesiveness and strength of the binary wax-based oleogels (Tavernier et al., 2017).

In this work, we evaluate the physical and textural properties of different binary mixtures of natural waxes at concentrations of less than 4% (w/w) in olive oil to minimize flavor issues related to the undesirable flavor of waxes. In the process, we discover a synergistic effect between specific waxes, which allows for the manufacture of an olive oil oleogel containing low amounts of wax but similar rheological properties to those of a commercial margarine.

## 2 Materials and methods

### 2.1 Sample preparation

Four natural waxes, SFW, RBW, CDW, and BW, were obtained as a gift from Koster Keunen Inc. (Watertown, CT, USA). Refined, bleached, and deodorized olive oil used in this study was purchased from the local market (Gallo, UNICI Inc., Concord, Canada). The fatty acid composition of olive oil was palmitic acid (13.1%), stearic acid (2.8%), oleic acid (72.7%), linoleic acid (8.6%), and other fatty acids (2.8%). The free fatty acid content of olive oil was 0.084 ± 0.013%, and the peroxide value was 3.4 ± 0.2 meq/kg olive oil. Different concentrations of binary wax mixtures containing 2 to 4 wt% of the various binary wax blends (1:1, 1:3, and 3:1 w/w) were prepared. Binary blends of waxes in olive oil were prepared by heating mixtures in an incubator at 100 °C for 60 min to dissolve the waxes and eliminate any effects of wax crystal memory. After heating olive oil, the peroxide value increased slightly to 5.5 ± 0.6 meq/kg olive oil.

The wax blends in olive oil were mixed with a glass stir rod and transferred to glass tubes, and allowed to gel at room temperature (22 °C). All samples were prepared at least in triplicates (n = 3), and stored for a minimum of 48 h prior to each analysis. To compare the physical and functional properties of binary wax mixtures in olive oil, a commercial soft margarine was purchased at a local supermarket (Imperial Margarine, Unilever Canada, Toronto).

### 2.2 Differential scanning calorimetry (DSC)

Melting points of the samples were obtained using a DSC model Q2000 (TA Instruments, Mississauga, ON, Canada). Nitrogen was used as the purge gas with a flow of 18 mL/min. Melting points (endothermic peak) of samples (10–15 mg) were determined by heating samples from 20 to 100 °C at the heating rate of 5 °C/min.

### 2.3 Powder X-ray diffraction

The crystal structure and polymorphic form of individual waxes and wax mixtures in olive oil were analyzed using a powder X-ray diffractometer (Multiflex Powder XRD spectrometer, Rigaku, Tokyo, Japan). The copper X-ray tube (wavelength of 1.54 Å) was operated at 40 kV and 44 mA. The measurement scan rate was set at 0.1°/min in the range 2θ = 1–30° at 20 °C. Peak positions were determined using MDI Jade 9 (MDI, Livermore, CA, USA) software.

### 2.4 Large deformation/plasticity

**Fig. 1:**
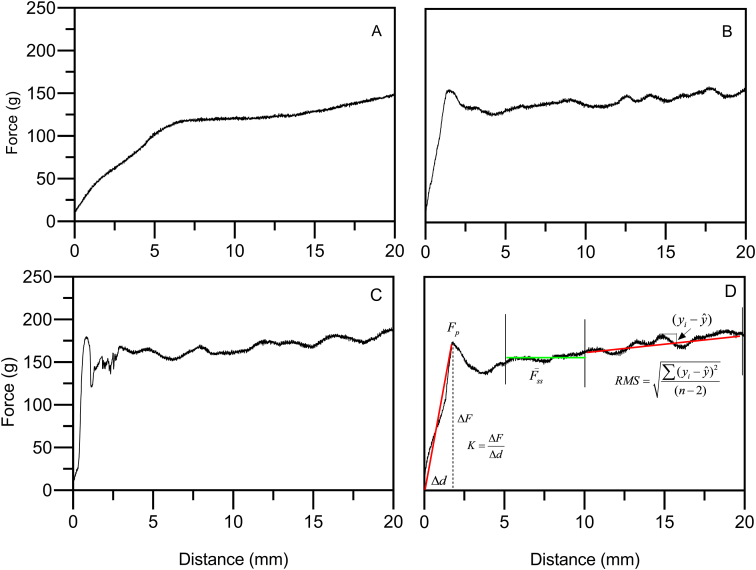
Back-extrusion force-penetration curves for (A) soft margarine, and 2.5% w/w wax oleogels in olive oil for (B) 1:3 (w/w) SFW-RBW, (C) 1:3 (w/w) BW-RBW, and (D) 3:1 (w/w) SFW-RBW. Indicated in panel D are the definitions of the parameters determined by back-extrusion.

The large deformation properties of the wax oleogels were assessed using the back extrusion analysis described in Gravelle et al. (2017). Binary wax oleogels were prepared as described above, and after complete melting, samples were transferred into 15 mL glass tubes and stored for 2 day at room temperature (22–23 °C) prior to analysis. The back extrusion test was performed using a TA. XT2 texture analyzer (Stable Micro Systems Ltd., Texture Technologies Corp., Scarsdale, NY, USA) equipped with a 30 kg load cell and a cylindrical stainless steel probe (height = 89 mm; diameter = 9.2 mm) with a truncated semi-spherical tip (height = 6.75 mm; diameter = 10.2 mm). Samples were penetrated to a depth of 20 mm at a speed of 1.5 mm^−1^ at room temperature. The relative plasticity of the gels was evaluated based on the profile of the flow curve once steady-state flow was achieved, between 10 and 20 mm penetration, by calculating the root mean square deviation from a linear regression of the steady-state region between 10 and 20 mm back extrusion penetration (Gravelle et al., 2017). Also, the flow behavior of the wax oleogels was compared to that of commercial soft margarine. The commercial soft margarine was warmed to room temperature and stuffed into 15 mL glass tubes to test at room temperature. The force constant of the oleogels was calculated by dividing the initial peaks’ breaking force by the deformation at that point. The steady-state back-extrusion force was determined as the average force between 5 and 10 mm penetration, where the force was mostly constant. Different ways of calculating these parameters was attempted and this strategy was designed from empirical observations of system behavior. Examples of different back-extrusion force deformation curves are shown in Fig. 1, as well as the way in which mechanical parameters were defined and calculated.

### 2.5 Polarized light microscopy

The microstructure of wax oleogels was determined using an optical microscope model BX60 (Olympus Optical Co., Tokyo, Japan) equipped with a 20X objective lens. Images were captured (20X) using a digital camera model DP71 (Olympus Optical Co., Tokyo, Japan) and an Olympus v1.0 cellSens software. A 5-μL of molten wax oleogels was placed on a preheated (80 °C) glass microscope slide and covered with a preheated (80 °C) glass coverslip. Then the slides were heated at 80 °C for 15 min to erase the crystal memory, then cooled to 22 °C and held for ten days prior to optical analysis.

### 2.6 Oil loss

To calculate the amount of released oil from the oleogel structures, first the weights of empty Eppendorf tubes were determined (a). For each sample, about 1 mL of completely melted wax oleogel was placed into the pre-weighed Eppendorf tubes, and allowed to crystallize for 2 day at room temperature to complete the crystallization process, and the tube weight recorded (b). Tubes were then centrifuged at 14,000 rpm for 30 min at room temperature. The tubes were then turned over and left overnight on a filter paper, allowing the drainage of the separated oil, and the filter paper weighed (c). The oiling-off (%) was calculated using the following equation:

-a) Oil Loss (%) = 100 x [(b-a) - (c-a)] / (b

### 2.7 Fatty acid composition and quality characteristics of olive oil

Olive oil fatty acid methyl esters were prepared based on the method developed by Christie (1982). An Agilent 6890-series gas chromatograph (Agilent Technologies, Inc., Wilmington, DE, USA) with a 7683-series auto-sampler was used to define the fatty acid composition of the olive oil. A 60 m × 0.22 mm internal diameter with a 0.25 μm film thickness GC column BPX70 (SGE Inc. Austin, TX, USA) was used. The oven temperature was programmed to increase from 110 to 230 °C (4 °C/min) and was maintained at 230 °C for 18 min. The injector temperature was set at 250 °C and operating at 20.1 psi with a flow of 17.7 mL/min. Helium, a carrier gas, flowed at an average velocity of 25 cm/s. A flame ionization detector was set at 255 °C with 450 mL/min air and 50 mL/min helium flowing. The GC separation peaks were analyzed using Open LAB software (Agilent Technologies). The fatty acid composition was determined by comparing the retention times of the peaks to internal standards.

The free fatty acid content (% w/w) and peroxide value (meq/kg oil) of olive oil used in this study were determined according to the American Oil Chemist's Society Official Methods of analysis for free fatty acids (Ca 5a-40) and peroxide value (Cd 8b-90).

### 2.8 Image analysis

Brightfield images were analyzed using ImageJ (Rasband, W.S., ImageJ, U. S. National Institutes of Health, Bethesda, Maryland, USA, https://imagej.nih.gov/ij/, 1997–2018). Images were transformed to 8-bit format, followed by Background Subtraction, Inversion (to make background black and features white), and Auto Thresholded using the Default option. The Box-counting Fractal Dimension was then determined using the standard algorithm under Tools using the default box sizes 2,3,4,6,8,12,16,32,64, and specifying a Black Background. Three images for each wax combination acquired using 10x, 20x and 40x objective lenses, were analyzed and the mean and standard error reported. For the estimation of crystallite size, we used the same thresholded and inverted images collected with the 10x objective lens, and used the Analyze Particles function to estimate crystallite size. We first Set Scale to 9.6436 μm per pixel and estimated the size of the crystals in the images bounded between 1 and 1000 μm^2^. This provided a balance between resolution and sensitivity.

### 2.9 Statistical analysis

The statistical analysis was carried out using GraphPad Prism software version 5.0 (La Jolla, CA, USA). All analyses were run at least in triplicates, and results were stated as mean values ± standard deviations. Data were evaluated using one-way ANOVA (Tukey's test), and the probability of *p* < 0.05 was considered significant.

## 3 Results and discussion

### 3.1 Oil loss

The oil-binding capacity of wax oleogels is one of the main characteristics that define their functionality in food products. Waxes should provide a crystalline structure able to tolerate processing conditions involving mixing and shearing while not falling apart and releasing all the bound oil. Many intrinsic factors have an effect on oil binding capacity in wax oleogels, including solid-state crystal structure, crystal size, and shape, as well as their spatial distribution and order (Marangoni, 2002). All of these will be determined by the chemical composition of the system as well as external fields. Previous studies showed the oil binding capacity of wax oleogels is highly related to chemical composition and the presence of minor components (impurities) in waxes. The structural properties, size of wax crystals, and spatial distribution of the wax network also have an effect on the oil-binding capacities of wax oleogels (Blake et al., 2014).

In this study, oil binding capacity was determined by exposing the oleogels to a centrifugal force for a specific time-temperature combination and measuring the amount of oil released gravimetrically. Some of the binary wax mixtures displayed a high oil-binding capacity. For example, the addition of 3% of RBW-SFW or 3% of CDW-BW to olive oil at different proportions was sufficient to eliminate oil losses. Moreover, similar results were obtained for binary mixtures of BW-RBW (1:3 w/w) and CDW-RBW (1:3 w/w). These results could be interpreted as a synergistic effect of specific waxes in olive oil (Blake et al., 2018). In 2021, Shi et al. showed a zero oil loss for a 5% binary wax oleogel containing BW-ZAW (75:25 w/w) binary mixture. The average oil loss for 3% (w/w) of individual and binary mixtures of natural waxes in olive oil is shown in Table 1.

**Table 1:** Oil Loss for 3% neat and binary mixtures of sunflower wax (SFW), rice bran wax (RBW), candelilla wax (CDW), and beeswax (BW) in olive oil. Values represent the means and standard deviation of n = 3 replicates.*

Oil loss (% w/w) in pure waxes			
CDW	23.50 ± 2.12^b^	SFW	0^a^
RBW	0^a^	BW	28.17 ± 2.47^c^
Oil loss (% w/w) from wax blends in olive oil (1:1 w/w ratio)			
RBW-SFW	0^a^	RBW-CDW	17.04 ± 1.42^d^
SFW-CDW	58.49 ± 0.70^b^	RBW-BW	10.99 ± 0.33^e^
SFW-BW	5.30 ± 0.07^c^	CDW-BW	0^a^
Oil loss (% w/w) from wax blends in olive oil (1:3 w/w ratio)			
SFW-RBW	0^a^	RBW-CDW	25.37 ± 0.71^d^
SFW-CDW	10.34 ± 0.79^b^	RBW-BW	0.94 ± 0.45^a^
SFW-BW	15.09 ± 0.52^c^	CDW-BW	0^a^
Oil loss (% w/w) from wax blends in olive oil (3:1 w/w ratio)			
SFW-RBW	0^a^	RBW-CDW	0^a^
SFW-CDW	28.26 ± 0.34^b^	RBW-BW	0^a^
SFW-BW	1.02 ± 0.54^c^	CDW-BW	0^a^

*Different superscript letters for each wax blend ratio represent statistically significant differences(P < 0.05) in oil loss between samples.

### 3.2 Hardness

The hardness of a wax oleogel is one of the main factors to determine the potential applicability of the wax oleogels, in particular for the potential of the wax oleogels in formulating products mimicking margarines, shortenings, and spreads (Tavernier et al., 2017). In this study, the back-extrusion steady-state force (Table 2) and elastic constant (Table 3) were used as an index of the oleogel hardness.

**Table 2:** Steady-state back extrusion force (g-force) of olive oil wax oleogels structured using a total amount of wax of 2%, 2.5%, 3%, 3.5% and 4% (w/w) in olive oil. The wax contained either individual or binary mixtures of SFW, RBW, BW and CDW at different mass ratios. Values represent the means and standard deviation of n = 3 replicates.*

Binary blend ratios (w/w)	Total percentage of wax in olive oil (% w/w)				
	2.0%	2.5%	3.0%	3.5%	4.0%
SFW-RBW 1:0	51.9 ± 16.5^a^	75.5 ± 29.9^a^	115.1 ± 34.1^a^	157.6 ± 24.1^a^	198.4 ± 10.9^a^
SFW-RBW 3:1	135.6 ± 15.1^b^	158.6 ± 16.5^b^	211.8 ± 25.5^b^	237.4 ± 34.1^b^	270.1 ± 30.2^b^
SFW-RBW 1:1	122.7 ± 11.6^b^	179.3 ± 5.7^b^	204.6 ± 11.5^b^	270.8 ± 16.9^b^	296.9 ± 40.6^b^
SFW-RBW 1:3	128.5 ± 12.3^b^	189.4 ± 11.9^b^	244.9 ± 20.5^b^	265.9 ± 25.3^b^	321.3 ± 27.3^b^
SFW-RBW 0:1	108.7 ± 16.5^b^	148.8 ± 19.6^b^	204.7 ± 17.2^b^	227.1 ± 27.3^b^	288.3 ± 16.7^b^
Blends ratios	2.0%	2.5%	3.0%	3.5%	4.0%
CDW-BW 1:0	24.5 ± 1.0^a^	34.3 ± 3.4^a^	49.2 ± 3.8^a^	64.3 ± 3.4^a^	114 ± 21.2^a^
CDW-BW 3:1	30.53 ± 16.6^a^	42.2 ± 16.8^a^	58.9 ± 16.2^a^	85.8 ± 11.7^a^	117.8 ± 14.4^a^
CDW-BW 1:1	74 ± 11.0^b^	131.5 ± 33.1^b^	179.6 ± 32.9^b^	194.2 ± 47.5^b^	222.2 ± 40^b^
CDW-BW 1:3	31.14 ± 15.0^a^	40.3 ± 22.2^a^	61.9 ± 32.7^a^	77.7 ± 34.4^a^	144.3 ± 22.1^a^
CDW-BW 0:1	11.24 ± 2.6^a^	20.1 ± 6.6^a^	25.8 ± 12.2^a^	47.8 ± 12.8^a^	59.5 ± 21.8^a^

*Different superscript letters in the same column indicate statistically significant differences (*P<0.05*) in hardness.

**Table 3:** Apparent elastic constant (g force/mm) of wax oleogels structured using 2–4% (w/w) of individual and binary mixtures of SFW, RBW, CDW and BW in olive oil. Values represent the means and standard deviation of n = 3 replicates.*

Binary blend ratios (w/w)	Total percentage of wax in olive oil (w/w)				
	2.0%	2.5%	3.0%	3.5%	4.0%
SFW-RBW 1:0	54.9 ± 9.4^a,A^	67.2 ± 9.1^a,A^	63.4 ± 9.7^a,A^	87.8 ± 36.4^a,b,A^	120.1 ± 34.1^b,A^
SFW-RBW 3:1	93.6 ± 56.1^a,A,B^	179.8 ± 55.2^a,B^	203.8 ± 70.8^a,B^	213.4 ± 46.7^a,B^	147 ± 48.4^a,A^
SFW-RBW 1:1	143.7 ± 57.9^a,B^	158.3 ± 30.4^a,b,B^	204.2 ± 82.7^a,b,B^	144.6 ± 15.9^a,A,B^	263.8 ± 46.4^b,B^
SFW-RBW 1:3	105.2 ± 1.4^a,A,B^	241.7 ± 45.8^a,B,C^	297.1 ± 16.1^a,B^	195.3 ± 70.0^a,B^	302.1 ± 21.5^a,B^
SFW-RBW 0:1	45.9 ± 14.4^a,A^	60 ± 19.6^a,A^	87 ± 27.1^a,b,A^	132.3 ± 37.3^b,A,B^	138.4 ± 14.2^b,A^
Binary blends	2.0%	2.5%	3.0%	3.5%	4.0%
CDW-BW 1:0	11.4 ± 2.4^a,A^	26.8 ± 3.1^b,A^	65.2 ± 10.5^c,A^	36.9 ± 7.6^b,A^	67.3 ± 5.0^c,A^
CDW-BW 3:1	21.5 ± 6.3^a,A^	42.4 ± 13.8^a,b,c,A,B^	48.3 ± 11.3^b,c,A^	62.4 ± 7.8^c,d,A^	79.3 ± 16.8^d,A,B^
CDW-BW 1:1	78.4±2^a,B^	104.9 ± 12^a,B^	123.7 ± 43.4^a,B^	101.2 ± 37.6^a,A^	133.6 ± 24.1^a,B^
CDW-BW 1:3	25.8 ± 6.5^a,A^	43.9 ± 6.2^a,c,A,B^	101 ± 23^b,d,A^	62.9 ± 14.9^c,d,A^	86.2 ± 7.1^d,A,B^
CDW-BW 0:1	10.7 ± 0.7^a,A^	9.7 ± 4.7^a,A^	12.6 ± 1.3^a,A^	26.8 ± 3.9^b,c,A^	36.1 ± 9.2^c,A^

*Different lowercase superscript letters in the same row show statistically significant differences (*P<0.05*) in elastic constant as a function of differences in total wax concentration, while uppercase superscript letters in the same column represent statistical differences (*P<0.05*) in the elastic constant between wax ratios at a fixed concentration.

Statistical analysis showed that the steady-state back-extrusion force of RBW-SFW mixtures in olive oil at all concentrations was significantly (*P* < 0.05) higher than the hardness of 100% SFW oleogels. However, no significant differences were observed between blends and 100% RBW (Table 2). For CDW-BW oleogels in olive oil at all total wax concentrations, only the 1:1 (w/w) binary mixture showed a significant difference (*P* < 0.05) relative to other wax mixtures (1:3 and 3:1) and individual waxes (Table 2). Moreover, we found that olive oil wax oleogels containing certain binary mixtures of SFW-RBW and CDW-BW displayed an elastic constant relative to the individual wax oleogels (Table 3). Based on these results, a synergistic effect was proposed for these specific binary mixtures of waxes. This synergistic effect could be due to the chemical composition of binary wax mixtures. Wax esters are the main components in SFW (96–100%) and RBX (92–97%), while this amount for BW and CDW were significantly lower (58–71%) and (16–35%), respectively, that shows the diversity of different components in these waxes (Blake et al. (2014). In a recently published paper, Brykczynski et al. (2022) showed a significant increase in G* in binary wax esters (1:1 w/w ratio) containing similar total carbon number wax esters, suggesting some sort of “compound” formation, or synergistic interaction at the molecular level. Previous studies showed the total carbon number for RBW and SFW were (44–64) and (36–54), respectively (Blake et al. (2014)). These could then fall within the compound formation range suggested by Brykczynski et al. It is also noteworthy to mention that while 2.5% BW and CDW yielded a very weak gel (less than 50 g-force), the steady-state back-extrusion force of the 2.5% CDW-BW 1:1 ratio oleogel was similar to that of a commercial soft margarine (134.88 ± 17.29 g-force). Similarly, Winkler-Moser et al. (2019) showed that oleogels containing binary mixtures of CDW-BW were significantly firmer than pure CDW and BW oleogels. Moreover, in 2020, Hwang and Winkler-Moser showed margarine analogs that were made with 3% binary wax mixtures of CDW-BW (1:3) had higher firmness than those made with the individual waxes.

Tavernier et al. (2017) suggested that the formation of RBW crystals in binary wax mixtures could act as a backbone for the crystallization of the BW and the development of a hybrid wax network. Tavernier et al. (2017) showed the binary mixtures of 5% wax oleogels containing 4% BW:1% RBW and 3.5% BW:1.5% RBW showed a significantly greater hardness than 5% BW and 5% RBW. The authors attributed the higher hardness of binary wax mixtures to polymorphic transitions of the BW crystal network that was developed upon storage.

### 3.3 Plasticity/brittleness

The relative plasticity of waxes and their binary blends in olive oil were evaluated using back extrusion flow curves. The back extrusion technique can be used to quantify and show the brittleness and plastic flow behavior of materials (Gravelle et al., 2017). In this technique, the downward movement of the probe and its penetration into the gel structure causes gel flow in the opposite direction of the probe. We have previously shown that the noise in the steady-state region of the flow curve (the flatter portion, post-yield, and flow) is related to the relative plasticity and brittleness of the oleogel. The brittleness indices of the individual waxes and their binary mixtures in olive oil (2–4% w/w) are shown in Table 3. In general, with an increasing total wax concentration in olive oil from 2% to 4%, the plasticity decreases, and the brittleness increases, as indicated by a higher brittleness index. (Table 4).

**Table 4:** Brittleness index of binary oleogel structured using 2–4% (w/w) of SFW, RBW, CDW, and BW in olive oil. Values represent the means and standard deviation of n = 3 replicates.*

Binary blend ratios (w/w)	Total percentage of wax in olive oil (w/w)				
	2.0%	2.5%	3.0%	3.5%	4.0%
SFW-RBW 1:0	2.86 ± 1.16^a,A^	4.29 ± 1.51^a,b,A^	6.17 ± 2.73^a,b,A^	5.34 ± 1.62^a,b,A^	7.28 ± 2.55^b,A^
SFW-RBW 3:1	3.03 ± 0.88^a,A^	4.65 ± 1.34^a,b,A^	9.96 ± 1.84^b,c,A^	11.47 ± 2.97^c,A^	11.46 ± 5.02^c,A^
SFW-RBW 1:1	2.60 ± 0.74^a,A^	4.27 ± 0.11^a,A^	5.15 ± 1.65^a,A^	5.52 ± 1.12^a,A^	13.60 ± 5.16^b,A^
SFW-RBW 1:3	3.58 ± 0.85^a,A^	3.39 ± 0.30^a,A^	3.91 ± 0.24^a,A^	7.45 ± 1.92^b,A^	8.81 ± 1.38^b,A^
SFW-RBW 0:1	1.92 ± 1.04^a,A^	2.81 ± 0.70^a,b,A^	5.69 ± 3.02^a,b,A^	4.54 ± 1.42^a,b,A^	7.36 ± 3.43^b,A^
Binary blends ratios	2.0%	2.5%	3.0%	3.5%	4.0%
CDW-BW 1:0	1.43 ± 0.21^a,A^	1.61 ± 0.40^a,A^	2.17 ± 0.48^a,A^	2.98 ± 1.08^a,A^	3.2 ± 2.06^a,A^
CDW-BW 3:1	2.53 ± 3.43^a,A^	2.01 ± 1.55^a,A^	4.02 ± 4.21^a,A^	5.18 ± 2.19^a,A^	7.98 ± 2.82^b,A^
CDW-BW 1:1	8.26 ± 1.16^a,B^	18.54 ± 7.53^b,B^	15.87 ± 7.12^b,B^	17.77 ± 3.86^b,B^	20.26 ± 11.56^b,B^
CDW-BW 1:3	2.01 ± 1.51^a,A^	2.93 ± 2.80^a,A^	4.05 ± 2.73^a,A^	5.74 ± 3.00^a,b,A^	11.52 ± 4.25^b,A,B^
CDW-BW 0:1	0.76 ± 0.00^a,A^	1.02 ± 0.14^a,A^	0.92 ± 0.23^a,A^	2.17 ± 1.17^b,A^	2.79 ± 1.39^b,A^

*Different lowercase superscript letters in the same row show statistically significant differences (*P<0.05*) in brittleness index as a function of differences in total wax concentration, while uppercase superscript letters in the same column represent statistical differences (*P<0.05*) in brittleness index between wax ratios at a fixed concentration.

The lowest brittleness indices were observed for 4% wax oleogels structured using CDW-BW 1:1 (w/w) and CDW-BW 3:1 (w/w). A lower brittleness (higher plasticity) combined with a relatively high firmness is desirable to mimic the functionality of margarine and spreads. The brittleness of soft margarine used in this study was 3.4, which is similar to that of 2.5% and 3% binary mixtures of SFW-RBW (1:1, 3:1, and 1:3) and CDW-BW (1:3). Interestingly, the 4% binary mixture of BW-RBW 1:3 in olive oil showed a brittleness index of 4, which was the lowest value of all the other blends at that concentration. Based on these results, the addition of 2–3% binary mixtures of waxes to olive oil was enough to provide a three-dimensional crystal structure with plasticity similar to that of soft margarine. Statistical analysis showed no significant differences (p < 0.05) between the brittleness of oleogels made with different ratios of SFW-RBW mixtures. While this difference for the 1:1 binary mixture of CDW-BW compared to individual CDW and BW oleogels was significant (Table 4). Jana and Martini (2016b) reported that the storage modulus (G’) of oleogels was higher for a binary mixture of RBW-SFW (20%, 50%, and 80% w/w) in soybean oil than for 2.5% olegoels structured by individual SFW or RBW.

### 3.4 X-ray analysis

**Fig. 2:**
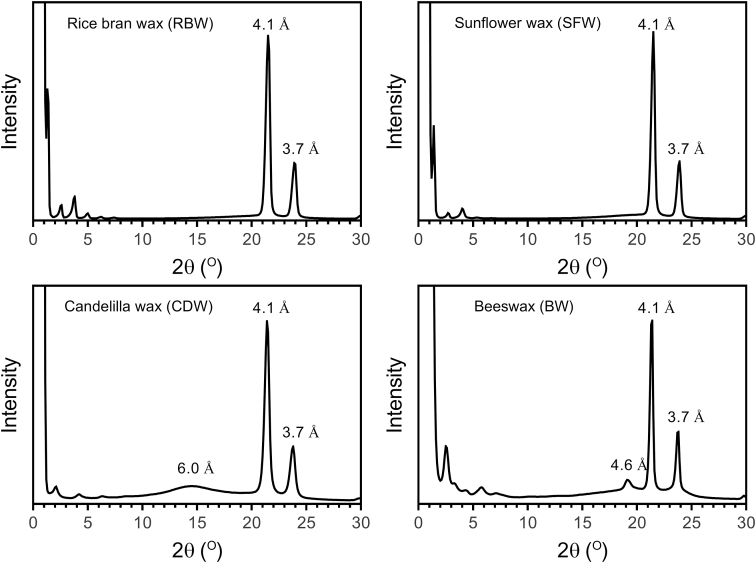
Small and wide-angle powder X-ray diffraction spectra of pure waxes.

**Fig. 3:**
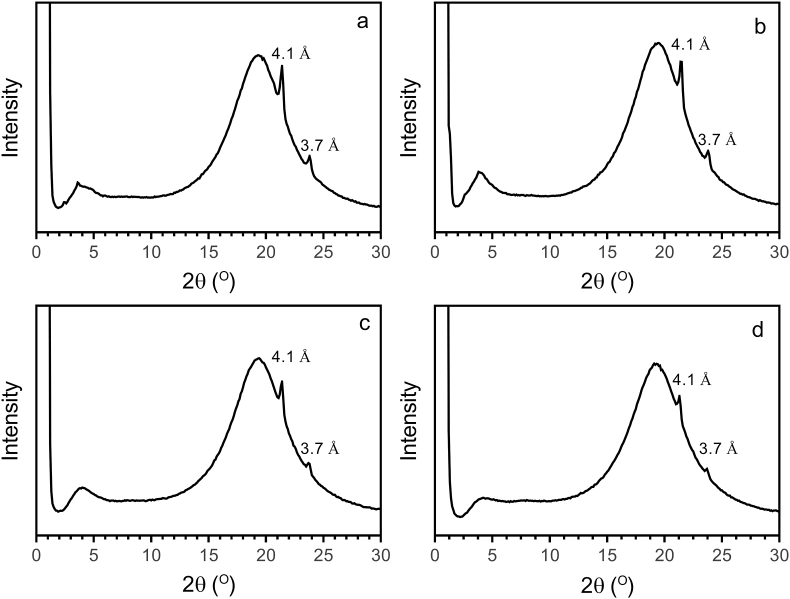
Small and wide-angle powder X-ray diffraction spectra of 3% (w/w) waxes in olive oil: (a) RBW, (b) SFW, (c) CDW and (d) BW.

Small and wide-angle powder X-ray diffraction spectra of neat RBW, SFW, CDW, BW, and their 3% (w/w) oleogel counterparts in olive oil are shown in Fig. 5, Fig. 6, respectively. In previous studies of plant wax crystal structures in the wide-angle region, two diffraction peaks at 4.1 Å and 3.7 Å have been reported (Blake et al., 2018; Dassanayake et al., 2009). In our study, for all neat waxes and their 3% (w/w) oleogels in olive oil, an identical wide-angle X-ray diffraction pattern was obtained (Fig. 2, Fig. 3).

The presence of two diffraction peaks at 4.1 Å and 3.7 Å correspond to orthorhombic perpendicular subcell packing arrangements. This crystal structure is similar to the β′ crystal polymorphic form of fats. Since the size of fat crystals in β′ polymorphic form is small, margarines and shortenings in β′ crystal form show smooth texture with high spreadability (Dassanayake et al., 2009). In our study, the “long spacing”, the SAXS reflection corresponding to the (001) plane of BW, CDW, RBW, and SFW in the β′ crystal form was at 69.95 ± 0.21 Å, 42.14 ± 0.23 Å, 69.32 ± 1.55 Å, 65.35 ± 0.70 Å, respectively. Based on the X-ray diffraction peaks in the SAXS region, CDW had the smallest lamellar size of the other three waxes. This structure can be addressed to the molecular composition of waxes (Blake et al., 2014). No dramatic differences were observed between the small and wide-angle powder X-ray diffraction spectra of 3% (w/w) RBW, SFW, CDW, and BW in olive oil (Fig. 3). Since all waxes used in this study were crystallized in an orthorhombic crystal polymorph, the authors believe that blending doesn't not have an effect on the crystal structure of wax mixtures.

### 3.5 Crystal morphology

**Fig. 4:**
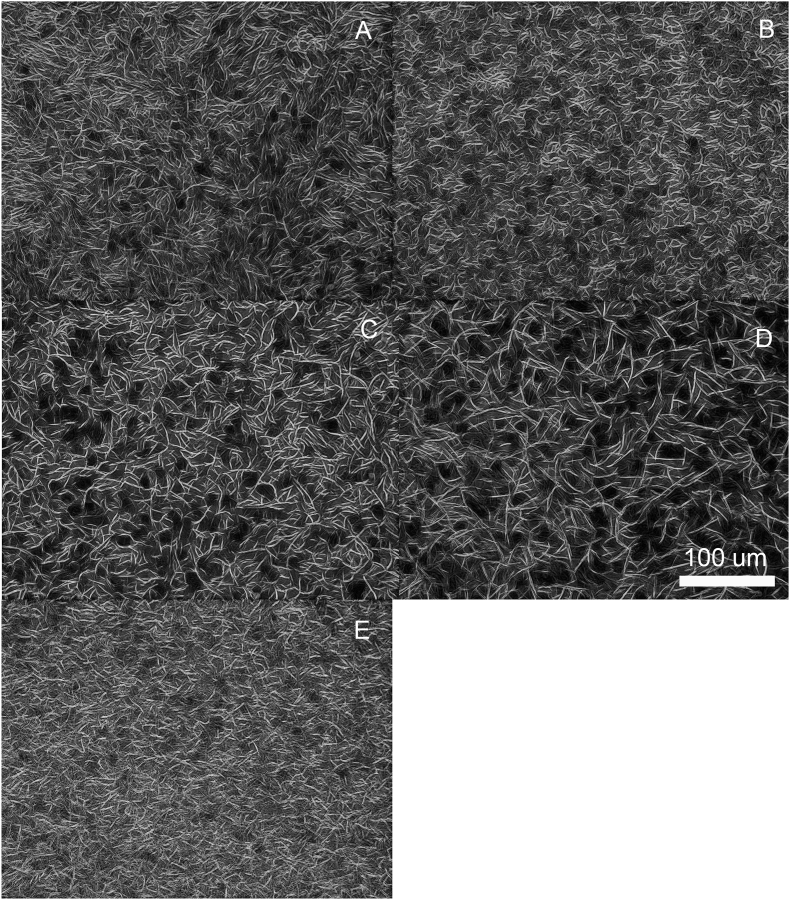
Inverted and background subtracted brightfield light micrographs of 3% (w/w) waxes in olive oil: (a) SFW, (b) RBW-SFW 1:3 w/w, (c) RBW-SFW 1:1 w/w, (d) RBW-SFW 3:1 w/w and (e) RBW.

Polarized light microscopy (PLM) is a very useful tool to study the crystal structure of wax oleogels. Upon cooling of binary wax mixtures, the wax crystallites start to make a network with properties that are highly dependent on the morphology and size crystalline wax components. The morphology of a wax oleogel crystal network mostly depends on the chemical composition of waxes (wax esters, fatty alcohols, free fatty acids, and hydrocarbons) and could be described as needle-shaped or spherical structures dispersed in liquid oil under polarized light microscopy as a thin film (Doan et al., 2017). The wax crystalline network growth could be the address to the strong van der Waals interactions between long wax-esters, hydrocarbon chains, and polar functional groups between lamellar planes (Fayaz et al., 2017). Light micrographs of 3% (w/w) SFW, RBW, and their blends (1:3, 1:1, and 3:1) in olive oil are shown in Fig. 4. RBW and SFW oleogels displayed needle-shaped and fibrous structures that have been considered the main reason for their efficient gelation properties (lower critical concentration and greater oil-binding capacity due to a higher gelation surface) (Yılmaz and Öğütcü, 2014; Blake et al., 2014). Qualitatively, our results suggest that blends of SFW and RBW displayed longer crystals than their individual wax oleogel counterparts. This may be the cause for their increased hardness and oil binding capacity.

**Fig. 5:**
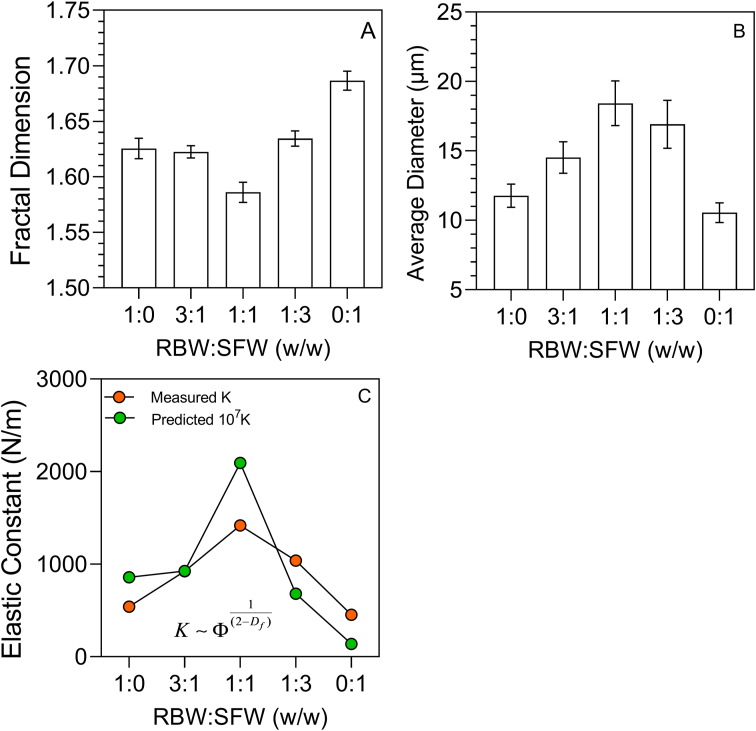
Image analysis parameters (A) box-counting fractal dimension, (B) average diameter (μm), and (C) elastic constant (N/m) of binary mixtures of RBW and SFW in olive oil. The elastic constant (N/m) data shown in panel (C) is for the 2% (w/w) wax mixtures in olive oil.

**Fig. 6:**
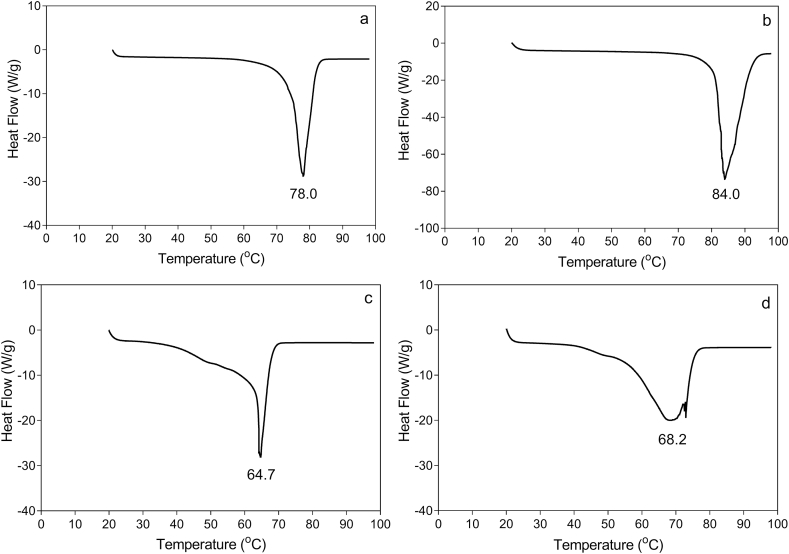
Differential Scanning Calorimetric melting profile of neat (a) SFW, (b) RBW, (c) BW, and (d) CDW.

### 3.6 Image analysis

One way to quantify the microstructure of any network is via the box-counting fractal dimension. This parameter is sensitive to the degree of fill of the embedding space and by the order of the mass distribution in space. Image analysis results using ImageJ showed a very clear depression in the box-Counting fractal dimension at 1:1 (w/w) RBW-SFW ratio relative to the neat waxes (Fig. 5A). This was due to a more heterogeneous distribution in crystalline mass and/or greater porosity. Another parameter that could be derived was the diameter of the crystals. An increase in crystal size was observed, with a maximum at 1:1 w/w RBW-SFX (Fig. 5B). This correlates well with visual observations of the micrographs. These data allowed us to estimate the elastic constant of the systems as predicted by Marangoni and Rogers (2003) and Marangoni (2000) for colloidal plastic disperse systems. A decrease in fractal dimension results in a higher elastic constant, explaining the observed increase in the measured elastic constant of the wax in oil mixtures (Fig. 5C). This increase in the elastic constant was controlled by the spatial distribution of mass in the wax crystal network and not the crystal size. As a matter of fact, an increase in crystal size would have resulted in a decrease in the elastic constant. The opposite was observed instead. Thus, the observed synergism between these waxes is due changes in the microstructure of the wax crystal network, induced by the blending the two waxes. Obviously, blending must cause changes in the kinetics of crystallization, which in turn lead to a lower fractal dimension and a coarser network with larger crystals. This may suggest a more sporadic nucleation and/or slower crystal growth, induced by the compositional changes.

### 3.7 Thermal properties

The DSC thermal analysis of pure SFW, RBW, BW, CDW and their binary mixture ratios (0:1, 1:3, 1:1, 3:1, and 1:0) at different total concentrations (2–4%) in olive oil are shown in Table 5 and Fig. 6. The highest melting point was obtained for RBW (84.12 ± 0.23 °C), followed by SFW (77.83 ± 0.30 °C), CDW (68.07 ± 0.23 °C), and BW (64.90 ± 0.25 °C).

**Table 5:** Differential Scanning Calorimetric melting points of SFW, RBW, CDW, BW and their individual and binary mixtures in olive oil. Values represent the means and standard deviation of n = 2 replicates.

	Total percentage of wax mixtures in olive oil			
Binary blends ratios	2.5%	3.0%	3.5%	4.0%
SFW-RBW 1:0	65.48 ± 0.35	66.19 ± 0.38	67.23 ± 0.96	67.35 ± 0.56
SFW-RBW 3:1	64.05 ± 0.09	64.92 ± 0.37	65.53 ± 0.35	66.36 ± 0.01
SFW-RBW 1:1	63.31 ± 0.34	63.42 ± 0.17	64.44 ± 0.13	65.19 ± 0.00
SFW-RBW 1:3	61.57 ± 1.01	62.41 ± 0.42	63.38 ± 0.30	63.42 ± 0.24
SFW-RBW 0:1	59.79 ± 0.57	60.73 ± 0.52	61.20 ± 0.06	61.62 ± 0.40
BW-CDW 1:0	46.37 ± 0.52	48.66 ± 0.26	48.80 ± 0.32	49.29 ± 0.62
BW-CDW 3:1	46.28 ± 0.12	47.73 ± 0.56	46.71 ± 0.08	48.90 ± 0.01
BW-CDW 1:1	44.64 ± 0.78	45.58 ± 0.53	45.99 ± 0.45	46.24 ± 0.05
BW-CDW 1:3	42.28 ± 0.23	43.86 ± 0.81	44.96 ± 0.45	45.01 ± 0.14
CDW-BW 0:1	43.89 ± 0.14	43.20 ± 1.00	45.76 ± 0.44	44.07 ± 0.37

As shown in Fig. 6, a sharp and narrow melting peak was obtained for all waxes except CDW, which showed a wide thermogram. The diversity in the various components of CDW (free fatty acids, free fatty alcohols, and hydrocarbons) compared to the other types of waxes that mainly contain esters may be the root cause for the wide DSC melting profile for this wax. Among the four different waxes used in this study, SFW and RBW showed the highest melting points (77.83 ± 0.30 and 84.12 ± 0.23, respectively). C.D. Doan et al. (2017) showed the high melting point of SFW oleogels was related to long-chain wax-esters that form platelet crystals and have a strong gelling property. Dissolving waxes in olive oil caused a dramatic reduction in melting points for both individual waxes and their binary mixtures (Table 5). For instance, the melting point of pure RBW was 84.12 ± 0.23 °C. While after dilution in olive oil at 2.5%, 3%, 3.5%, and 4% concentrations, the melting temperature decreased to 65.48 ± 0.35 °C, 66.19 ± 0.38 °C, 67.23 ± 0.96 °C, and 67.35 ± 0.56 °C, respectively.

According to C. D. Doan et al. (2017), wax crystallization is initiated by the supersaturation of waxes in liquid oil, and the crystallization temperature is affected by the wax concentration. A drop in melting temperatures of waxes in liquid oil is due to a dilution effect. Similar results for the dilution effect and decreasing the melting points of different waxes in plant oils were reported by Blake et al. (2014) and Yılmaz and Öğütcü et al., 2015. Yılmaz and Öğütcü reported lower melting points for 3% (w/w) BW and 3% SFW in olive oil, 44.36 ± 0.23 °C and 58.26 ± 0.18 °C, respectively, than the results we obtained in this study, 48.66 ± 0.26 °C and 60.73 ± 0.52 °C, respectively (Table 5).

**Fig. 7:**
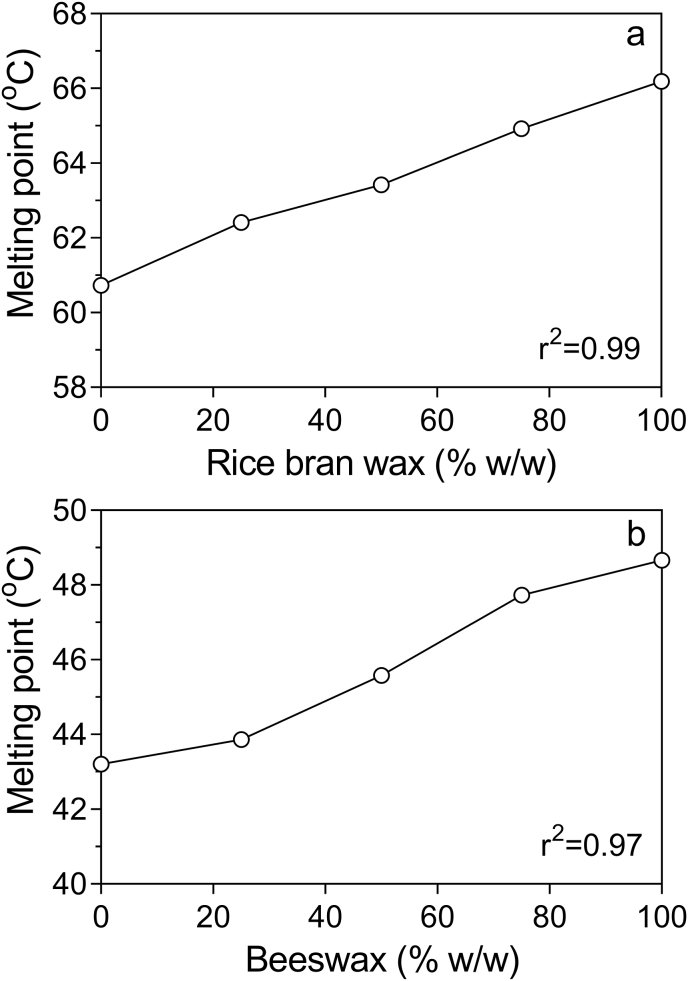
Binary melting diagrams of 3% w/w wax in olive oil: (a) RBW-SFW and (b) BW-CDW. The percentage stated in the x-axis refers to the relative percentage of one wax species relative to the other.

A mixing diagram for a 3% total concentration of RBW-SFW and BW-CDW mixtures at different ratios in olive oil (Fig. 7) showed an “ideal” mixing behavior between these waxes at different mass ratios. This pseudo phase diagram suggested high solid-state compatibility between SFW-RBW and BW-CDW blends in olive oil and a possible formation a mixed crystal between the synergistic waxes.

Jana and Martini (2016a) studied the phase behavior between SFW-RBW and CDW-BW mixtures. While they reported a high degree of compatibility for SFW-RBW mixtures, they showed a eutectic behavior and a drop in melting point for binary blends of CDW-BW. In the same year, Jana and Martini (2016b) reported a eutectic effect for mixtures of RBW-BW in soybean oil. However, this was not the case in our work, where complete compatibility in melting profiles of binary wax was observed. Hwang and Winkler-Moser (2020) reported a eutectic melting behavior for a margarine analog made with a 3% binary wax mixture of CDW-BW (1:1) at 43.85 °C. The melting points for margarines made with 100% CDW and 100% BW were 46 °C and 47.61 °C, respectively. They reported a constant decrease in dropping point with increasing BW proportions in binary mixtures, and no eutectic behavior was observed in the dropping point of binary mixtures.

## 4 Conclusion

In this study, we discovered a synergism between SFW and RBW, as well as between CDW and BW. These mixtures displayed an increased hardness, elastic constant, plasticity and oil binding capacity relative to wax oleogels structured by the same concentration of the individual waxes. Moreover, all wax oleogels displayed a β’ crystal structure. This synergism would allow for the use of less wax, thus improving the flavor of a spread manufactured using this oleogelation strategy, while also improving the economics of production. Based on the results, we recommend the use of 3% (w/w) wax oleogels structured using 1:1 (w/w) mixtures of SFW and RBW to manufacture edible spreads.

## Declaration of competing interest

The authors declare that they have no competing financial interests or personal relationships that could have appeared to influence the work reported in this paper.

## Author contribution

SMG designed experiments, carried out experimental work, analyzed the data and wrote the initial draft of the paper. SD analyzed experimental data. AGM obtained funding for the project, designed experiments, analyzed data, edited the manuscript.

## Declaration of competing interest

The authors declare that they have no known competing financial interests or personal relationships that could have appeared to influence the work reported in this paper.
